# Online Omics Platform Expedites Industrial Application of *Halomonas bluephagenesis* TD1.0

**DOI:** 10.1177/11779322231171779

**Published:** 2023-05-09

**Authors:** Helen Park, Matthew Faulkner, Helen S Toogood, Guo-Qiang Chen, Nigel Scrutton

**Affiliations:** 1 EPSRC/BBSRC Future Biomanufacturing Research Hub and BBSRC Synthetic Biology Research Centre SYNBIOCHEM, Manchester Institute of Biotechnology and Department of Chemistry, The University of Manchester, Manchester, UK; 2 Center for Synthetic and Systems Biology, School of Life Sciences, Tsinghua-Peking Center for Life Sciences, Tsinghua University, Beijing, China

**Keywords:** genomics, transcriptomics proteomics, principal components analysis (PCA), machine learning, *Halmonas bluephagenesis* TD1.0, polyhydroxyalkanoates (PHA), flagella, synthetic biology

## Abstract

Multi-omic data mining has the potential to revolutionize synthetic biology especially in non-model organisms that have not been extensively studied. However, tangible engineering direction from computational analysis remains elusive due to the interpretability of large datasets and the difficulty in analysis for non-experts. New omics data are generated faster than our ability to use and analyse results effectively, resulting in strain development that proceeds through classic methods of trial-and-error without insight into complex cell dynamics. Here we introduce a user-friendly, interactive website hosting multi-omics data. Importantly, this new platform allows non-experts to explore questions in an industrially important chassis whose cellular dynamics are still largely unknown. The web platform contains a complete KEGG (Kyoto Encyclopedia of Genes and Genomes) pathway enrichment analysis derived from principal components analysis, an interactive bio-cluster heatmap analysis of genes, and the *Halomonas* TD1.0 genome-scale metabolic (GEM) model. As a case study of the effectiveness of this platform, we applied unsupervised machine learning to determine key differences between *Halomonas bluephagenesis* TD1.0 cultivated under varied conditions. Specifically, cell motility and flagella apparatus are identified to drive energy expenditure usage at different osmolarities, and predictions were verified experimentally using microscopy and fluorescence labelled flagella staining. As more omics projects are completed, this landing page will facilitate exploration and targeted engineering efforts of the robust, industrial chassis *H bluephagenesis* for researchers without extensive bioinformatics background.

## Introduction

The volume of high-dimensional omics data is increasing rapidly as tools for collection and processing become streamlined. However, useful and comprehensive omics analysis remains a bottleneck in metabolic engineering.^1^ Omics data consist of proteomics, transcriptomics, and metabolomics data, which measure levels of protein, mRNA, and metabolite expression for cells under specific growth conditions. Taken independently, each of these datatypes is insightful, but a multi-layered approach shows cell dynamics more comprehensively. For example, multi-omics data have been successfully applied in the food industry to extend product shelf life, ensure microbial quality control, and improve the health content of products.^2^ These data have also been applied in the biomedical^1^ and biotechnology^3^ industries. Especially when paired with a well-curated reference genome, omics data will enhance our ability to determine causal relationships from cellular manipulations and help us reach standard industrial goals of improved production titre and cell growth.^3^

Using data to its full capacity is a challenge in and of itself. Computational tools are currently inadequate especially for biologists unfamiliar with data manipulation,^4^ and results are often used only for a single project or product. Lowering the effort and skill required for impactful insights is needed. Despite an increasing volume of data from high-throughput engineering pipelines, strain development often proceeds through trial-and-error without insight into complex cell dynamics.^5^ Classically, this can involve black-box random mutagenesis or directed evolution without mechanistic insight. Successful approaches include the ‘Design-Build-Test-Learn’ (DBTL) cycle which uses high-throughput screening to refine strain designs using iterative cell build stages.^6^ For example, one report mined proteomics data to guide the production of dodecanol in *E coli*.^7^ Such methods typically hinge on a single target compound and can be hindered for non-model organisms that lack a complete repertoire of engineering tools. A heavily designed strain may improve titre of a single product at the detriment of cell growth or other compounds synthesis and may only achieve high yields under a limited set of environmental conditions.^8^ Taken together, the engineering process involves considerable time and manpower, as seen in the key early sustainable biology successes story, the artemisinin pathway, which took more than 150 research years to complete.^7^

The speed and cost for omics data generation outpaces our ability to fully use and analyse data.^1^ Target-independent omics mining and cell morphology engineering are 2 noteworthy strategies to improve production. For example, white-box machine learning (ML) approaches allow insight into the inner workings of a model. Recently in *E coli* such an approach was developed using substantial omics data to unlock causal relationships in purine biosynthesis and antibiotic efficacy.^9^ A separate study developed 3 biofuel targets in parallel using omics data from 8 strains, drafting a computational workflow, including proteomics, metabolomics, and genome-scale modelling that can be used for future targets and strains.^3^ Meanwhile, mining of fluxomic and transcriptomic data were used to study the efficacy of 27 ML model prediction ability for 1143 *Saccharomyces cerevisiae* mutant phenotypes.^10^ Results from such studies are useful for cell engineering regardless of target but require extensive coding and machine-learning ability.

Approaches to improve strains using product-independent methods include manipulation of energy balance in the cell, outer membrane (OM) engineering, cell shape and morphology, and even the charge of cells.^11^ Changing the cell size can increase intercellular product accumulation. Cells with defective membranes can increase the oxygen and substrate permeability into the cell and increase their sensitivity to inducers or antibiotics.^12^ A recent study showed that lipopolysaccharide (LPS), Lipid A, O-antigen, and flagella genes disruption in *E. coli* all led to improved polyhydroxybutanoate (PHB) production.^13^ Meanwhile, deletion of all 76 flagella-related genes in *Pseudomonas putida* improved growth, decreased biofilming, and increased polyhydroxyalkanoate (PHA) production.^14^ In *H. bluephagenesis*, production gains have been realized from engineering the cell to (1) have a larger volume and length by the genetic removal of *mreB* and *ftsZ* genes^15^; (2) creating self-flocculating cells through an *etf* operon knockout^16^; (3) increasing oxygen availability through expression of bacterial *Vitreoscilla* haemoglobin (VHb) in the periplasm^17^; and (4) increasing the energy balance of reduced/oxidised nicotinamine adenine dinucleotide (NADH/NAD^+^) in the cell.^18^. These manipulations allow more product to fill the cell volume, which in turn decreases the separation cost of cell mass and products. In addition, it led to increased cell growth through enhanced oxygen uptake and improved flux towards products requiring high NADH usage in their synthesis. Finally, attempts have been made to manipulate the OM structure of *H. bluephagenesis*,^19^*E. coli*,^20^ and other bacteria such as *P. putida*,^14^ to characterize cell changes and increase product titres. These studies lay the groundwork to understand the complex OM, LPS, and flagellar assembly process. Moreover, they present a method to improve cell growth and overall productivity by freeing up energy through the removal of nonessential but energy-intensive membrane components.

Herein, this work addresses 2 major challenges facing metabolic engineering today by (1) easing the analysis process of high-throughput omics data and (2) expanding chassis industrial viability through target-independent means. Notably, our methodology allows researchers to explore cell dynamics without being constrained to one product target, as omics data focus on a largely unexplored but industrially important chassis. Most importantly, data have been published in a user-friendly website allowing researchers with no coding or data analysis background to draw conclusions and to design better strains. We validate the robustness of our platform’s multi-dimensional omics data through flagella exploration in *H. bluephagenesis*. Specifically, this newly developed site integrates proteomics and transcriptomics data, allows query into familiar websites such as KEGG, and facilitates exploration of the to be published HaloGEM cell flux model.

## Methods

### Omic analysis and website platform design

Initial omics datasets containing transcriptomic and proteomic data and genome-scale metabolic (GEM) model template used for analysis were obtained from our prior research (see ‘Availability of Data and Materials’). Data exploration was first performed in Jupyter notebook and commented copy of the workflow is available in GitHub, published along with additional analyses not yet transitioned to the web application. Key python packages used during development were scikit-learn,^21^ biopython,^22^ and cobrapy.^23^ Machine learning (ML) scikit-learn package was used for principal components analysis (PCA) decomposition and cluster heatmap. The biopython code was modified for KEGG mapping and pathway analysis, and GEM model flux variability analysis was performed using cobrapy. After analysis in Jupyter Notebook, useful components were transitioned to the web platform based on Python and HTML, using Dash.^24^ Dash is a Python library developed by Plotly Technologies Inc (https://plotly.com/dash/) with a focus on visualization and ML capabilities. Python graphs were transitioned to use Plotly to allow interactivity for users. The website link for the developed site is https://halomonas-td10-omics.herokuapp.com/apps/PCA and Jupyter Notebooks is https://github.com/helloftroy/Omics_TD1.0.

### Data preparation and PCA

PCA is a robust, pathway-independent and data-independent unsupervised ML technique that is standard practice for large data sets due to its ability to handle different datatypes.^25^ It can cleanly provide structure for complex omics data^26^ and its application can be used to guide engineering efforts in metabolic engineering.^27^ We used PCA as an orthogonal linear transformation to simplify big data sets while minimizing information loss. Each dataset (column in 14 column matrix) was first standardized in the conventional way: Z=(x−μ)/σ where *x* is the value, µ and σ are the mean and standard deviation of each column. Each individual column’s final distribution was checked through a histogram to make sure it was satisfactorily sigmoidal and that each column had comparable minimum and maximum. For the transformed analysis, the matrix was rotated via setting A1: (DNA_*i*_ + Protein_*i*_) and A2: (DNA_*i*_ – Protein_*i*_), for *i* = 1, 2, 3, 4, 5, 6, 7 for all 7 experimental conditions, resulting in 14 new columns. A1 is a magnitude-based calculation, enhancing the differences between DNA and protein. A2 is subtraction based, amplifying the differences seen between the omics type.

### Cell movement microscopy with cell tracking in Fiji and ImageJ

All chemicals and solvents were commercially sourced and were of analytical grade or better. Media components were purchased from Formedium (Norfolk, UK). The organism *H. bluephagenesis* TD1.0 was previously isolated from the Aydingkol Lake in Xinjian, China.^28^ Standard *H. bluephagenesis* cultivation was performed in an LB base (10 g/L tryptone; 5 g/L yeast extract) pH 9 with varying salt concentrations (LB20-LB100; LB base containing 20-100 g/L NaCl, respectively). Cultures (5 mL) in LB20, LB60, and LB100 were cultivated for 5 hours at 30°C with shaking, then diluted 10-fold into the same-salt concentration media. An aliquot (5 µL) of each was added to a microscope slide for imaging under bright-field NIKON Eclipse TE2000-U microscope. Four to five 20-second movies were taken of each slide at 0, 30, and 60 minutes, with more than 100 cells observed for each condition. Using the Fiji software, movies were processed using the following macro: Import movie, ‘Enhance Contrast’, ‘Remove Background’, change video to 8-bit, ‘Adjust Threshold’ (such that cells are highlighted against a black background). Cells were counted using ‘Analyse Particles’, with cell minimum size set to be 6 pixels, clicking ‘Show Outline’ and ‘Display Results’. This process also calculated particle size for morphology measurements. Finally, tracking of each particle (cell) distance was performed by using the tracking plugin ‘mtrack2’.

### NanoOrange fluorescence for flagella

NanoOrange was used to measure flagella via a fluorescence plate reader, using the manufacturer’s protocol with a few adjustments. Cultures (5 mL) were grown overnight in LB20, LB60, and LB100 broth. Cells from each media type were diluted 10-fold into LB20, LB60, LB100 in triplicate in two 24-well plates. After 3 hours incubation at 30°C with shaking, 200 µL aliquots were incubated for 10 minutes at 90°C with 5 µL NanoOrange working solution, then 200 µL aliquots were transferred into a 96-well plate. Fluorescence was measured using a ZEISS Axio fluorescence microscope after 5 minutes. Control samples were composed of sterile mediums incubated with NanoOrange. The fluorescence of the controls was subtracted from each culture data. Average values and standard deviation of triplicate samples were calculated.

## Results

### PCA reveals differentiated genes for experimental conditions

**Figure 1.**
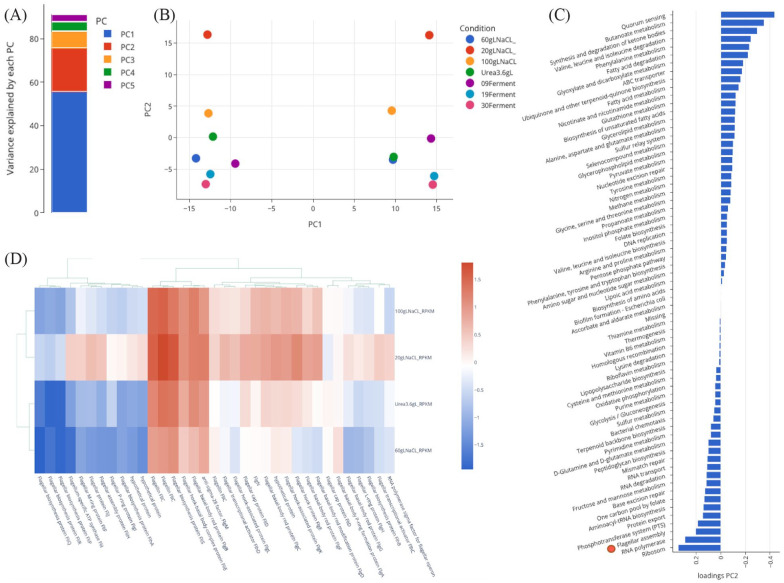
PCA and KEGG pathway analysis. (A) Variance explained by each PC. (B) Comparative PC analysis between PC1 and 2 over 7 experimental conditions. Two clear clusters are visible in PC1, separating proteomics and transcriptomics datasets, while conditions mirror each other along PC2. (C) KEGG pathway enrichment was performed by identifying genome annotations to KEGG pathways. The KEGG pathways are ranked using average loadings in PC2, enhancing differences between experimental conditions. Flagellar assembly is third from bottom with positive loading indicating flagellar variation drives differences seen in LB20 and LB100 (red circle). (D) Cluster heatmap for transcriptomic data with all annotated flagellar assembly genes. For other genes and conditions, please refer to the interactive web platform. ATP, adenosine triphosphate; PCA, principal components analysis; KEGG, Kyoto Encyclopedia of Genes and Genomes.

Both transcriptomic and proteomic data were used for the development of the interactive web platform taken from 7 experimental conditions: 3 fermentation samples (9-, 19-, 30-hour time points), and 4 shake flask experiments (LB20, LB60, LB100 [20, 60, and 100 g/L salt], and high urea [3.6 g/L urea]). Principal components analysis was performed to determine specific enzymes and metabolic pathways that drive variation between the 7 conditions. We found principal component 1 (PC1) explains 55% of the variance and that PC1 and principal component 2 (PC2) explains more than 75% of the variance, suggesting that our 14-column matrix can be simplified to 2 columns containing just PC1 and PC2 without substantial information loss (Figure 1A). A graph for PC1 against PC2 indicates that there are 2 distinct groups along the PC1 axis that cluster transcriptomic and proteomic data in 2 groupings (Figure 1B). This result was intriguing as the data were normalized before PCA (see ‘Methods’), suggesting there is a meaningful difference between transcriptomic and proteomic abundance. Meanwhile, PC2 appears to separate samples based on experimental condition, as the y-coordinates for PC2 are nearly identical across each experimental condition. We initially interpreted PC1 to measure differences between transcript and protein expression and PC2 to differentiate between experimental conditions regardless of data type.

The consistent pattern in PC1 and PC2 indicates the PCA approach is valid, so we performed a data transformation (rotation) for each condition to correct for any differences between proteomic and transcriptomic datatypes (Supplemental Figure S1). Specifically, we set A1 to be the addition of transcriptomic and proteomic columns, and A2 to be the difference between transcriptomic and proteomic columns, transformations that respectively enhance or ameliorate differences between datatypes. We found after rotation for all 7 experimental conditions that A1 and A2 cluster separately along PC1. However, the clusters along PC2 reveal A1 has a large range while A2 is tightly grouped near zero (Supplemental Figure S1). These results suggest there is little variation between conditions in terms of protein and mRNA expression (A2) and that these differences do not drive the variation between each experimental condition. This suggests that experimental-condition variations are enhanced, and any meaningful variation observed in transcription and translation likely exists in all conditions (A1). Specifically, in A1, LB20 was found to be extremely positive in PC2, LB100 was also positive, and other conditions were negative. The 30-hour fermentation results are most different from LB20. Readers can refer to the ‘PCA Analysis’ tab of the online platform to view and interact with PCA results (https://halomonas-td10-omics.herokuapp.com/apps/PCA).

### Pathway enrichment analysis integrate PCA loadings with the KEGG pathway database

**Figure 2.**
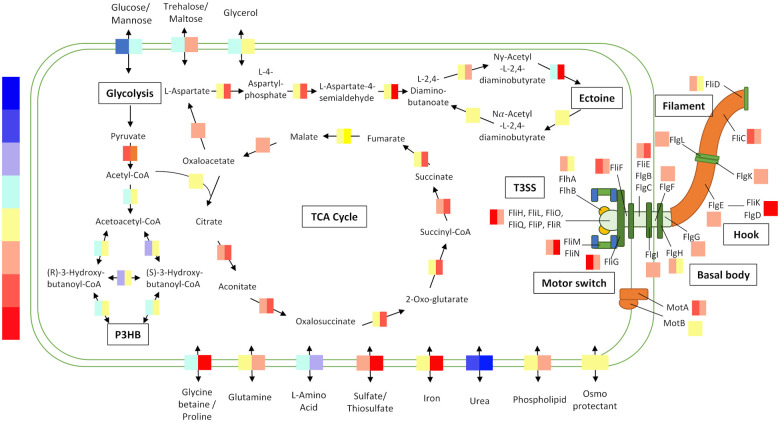
Transcriptomic overlay of key pathways. A combined view of key KEGG pathways using the web platform: butanoate metabolism (PH3B pathway), ectoine biosynthesis, TCA cycle, ABC transport, and flagellar assembly. The web platform was used to overlay omic data at different salt concentrations. Coloured boxes over arrows are LB20-LB60 (left) and LB100-LB60 (right) transcriptomic data. The scale bar (left) indicates expression, with blue and red the most underexpressed and overexpressed, respectively, and yellow/turquoise very similar to LB60. For other pathways, other experimental conditions, and proteomics results, please see the ‘KEGG Map’ section of the web platform. KEGG, Kyoto Encyclopedia of Genes and Genomes; TCA, tricarboxylic acid cycle; ABC, ATP-binding cassette.

*Halomonas bluephagenesis* has adapted to grow in a large range of salinities (10-200 g/L NaCl); however production and growth are usually optimal at 60 g/L.^19^ We were interested to explore the transcriptomic and proteomic expression to determine if any specific genes or pathways drive variation at different salinities. We used PC2, which previously showed clear separation between experimental conditions for the LB20, LB60, and LB100 datasets. When ranking genes with high positive PCA loadings in PC2, 7 out of the 10 highest loading genes were annotated to be flagellar assembly proteins or otherwise related to bacterial chemotaxis and motility (Supplemental Figure S1). Meanwhile, the most negative loadings in PC2 included many genes related to ATP-binding cassette (ABC) transport and quorum sensing. KEGG genome annotation was used for pathway enrichment to determine which areas of metabolism were most differentiated at different salinities (Supplemental Figure 1c). Notably, we saw the most variation between conditions were ribosomal proteins, RNA polymerase, flagellar assembly, quorum sensing, and butanoate metabolism (Figure 1c). Further investigation revealed expression in LB20 and LB100 mediums for these pathways was either greatly elevated or repressed compared with LB60 (Figure 2; ‘KEGG Map’ tab of online platform).

### Pathway enrichment for ABC transport, butanoate metabolism, ribosome, and quorum sensing

ABC transporters were among the top differentiated pathways between different salt conditions, with increasing salt leading to higher expression of ABC transporters (Figure 2). Specifically, the ectoine synthesis pathway is elevated in LB100 compared with LB60 and LB20, as well as glycine betaine, proline, glutamine, trehalose, and maltose transporters. These compounds are known osmolytes used by other halophiles to combat salt stress.^29^ Meanwhile, glucose and urea transporters were downregulated in both LB100 and LB20 compared with LB60, while sulfate/thiosulfate and iron transporters are upregulated in both salts and especially in LB100. High PC2 loading was seen in butanoate metabolism, a pathway of relevance to *H. bluephagenesis* because it is involved in synthesizing the valuable industrial target PHB. Specifically, omics data indicate these pathways are heavily downregulated in LB20 and are slightly upregulated in LB100 relative to LB60 (Figure 2). The loadings in PC2 were also noticeably enriched for RNA polymerase and ribosomal proteins, both of which were heavily upregulated compared with LB60, although overall protein abundance was similar between samples. Finally, quorum sensing was differentiated in PC2, and we found most quorum sensing genes upregulated in LB100 compared with LB60.

### Pathway enrichment for flagellar assembly and motility measurements at different osmolarities

**Figure 3.**
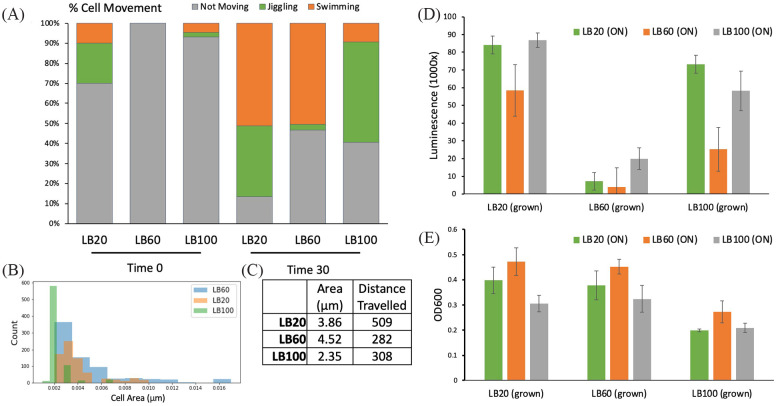
Initial cell motility, NanoOrange stain, and morphology measurements of *H. bluephagenesis* TD1.0. (A) Cell movement for *H. bluephagenesis* TD1.0 grown at different salt concentrations. Cultures were grown overnight (ON) in LB20, 60, and 100 (g/L NaCl). Cells were diluted into the same media and incubated for 3 hours, and videos were taken on a bright-field microscope (*t* = 0, 30, 60 minutes). Cell movement was counted for > 100 cells over 3 movies after movie processing in ImageJ/Fiji. (B) Histogram of nm area for each cell type after movies taken with microscope. There is a clear size difference between LB100 and LB60/LB20 cells. (C) The average pixel distance travelled and average nm area for cells in each movie after Fiji image processing. For average distance travelled, data were filtered to only measure moving cells. Both (B, C) data were taken using *t* = 30-minute movies, and averages were calculated over 3 movies (>100 cells.) (D, E) Flagella expression via luminescence using NanoOrange for *H. bluephagenesis* TD1.0 grown under different salt conditions. Cells were cultivated ON in LB20, 60, 100 (g/L NaCl). Each condition was diluted 1:10 into LB20, 60, 100 (grown). After 3 hours, cells were normalized to OD 0.2, and the flagella content was measured with NanoOrange stain using a 96-well microplate reader. (D) The fluorescence and (E) OD600 reading before normalization to OD 0.2. OD, optical density.

We found that the KEGG pathways for flagellar assembly and motility were both differentiated in our PCA and that flagella proteins were especially upregulated in both low-salt and high-salt conditions compared with LB60 (Figure 2). Flagellar assembly was selected for further study to experimentally verify whether flagella abundance correlated to our analytical findings. Cell motility of *H. bluephagenesis* TD1.0 was measured for LB20, LB60, and LB100 by taking movies on a bright-field microscope which were processed in ImageJ/Fiji to calculate the percentage of moving cells and the average distance travelled at each salinity. It was found that during growth in LB20, 87% of cells were swimming or otherwise motile compared with only 53% in LB60. In LB100, only 9% of cells were swimming, although 51% showed some non-Brownian motion (‘Jiggling’) without full swimming across the microscope slide (Figure 3A). For swimming cells, LB20-grown cells’ average distance travelled was 1.8 times greater than in LB60 medium, while LB100 and LB60-grown swimming cells had comparable distances travelled (Figure 3C). Using a NanoOrange stain for flagella, we measured the fluorescence of cells grown at different osmolarities. Cells grown overnight in LB20 showed consistently higher fluorescence staining than LB100 or LB60, with LB60 showing the lowest fluorescence of the 3 conditions (Figure 3D).

Interestingly, we noticed the morphology of cells varied under the bright-field microscope in media at different osmolarities. Specifically, the cell length of cells grown in LB100 were consistently very short (2.85 μm) while LB20 (3.85 μm) and LB60 (4.52 μm) showed comparable longer cell lengths (Figures 3B). We observed after 60 minutes LB100-grown cells had begun to form a biofilm, while cultivation in LB20 and LB60 showed no such phenomenon (Supplemental Figure S2 movie). After 60 minutes, the microscope coverslip began to dry, and we observed LB20 cells swimming quickly in the water away from the drying air bubble. In contrast, cells in LB60 medium showed no such directed swimming behaviour (Supplemental Figure S3 movie). Taken together, this suggests that cells in lower salt concentrations (LB20) are more motile, perhaps due to increased flagella. In contrast, high salinity cells (LB100) appear to have a smaller cell size with potentially increased flagella content and a higher tendency for biofilm formation than cells grown in LB60 cells.

### Genome annotation for flagella assembly and motility in H. bluephagenesis reveals genes in one sequential operon

Elucidation of the motility and assembly mechanism is desirable to properly select knockout targets that minimally tamper with the genome. Our analytical findings suggest that flagella knockouts would remove energy-intensive transcription, translation, and energy associated with flagella rotation at different salinities. However, the mechanisms for flagella assembly are complex and not fully characterized in *H. bluephagenesis*.

**Figure 4.**
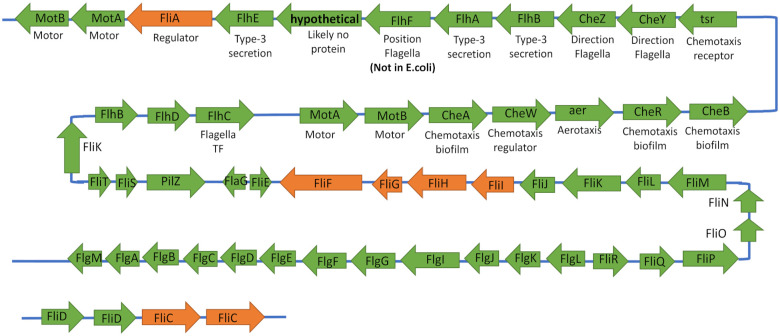
Annotated main flagella operon in *H. bluephagenesis* TD1.0 genome. Green-coloured genes are flagella-related and orange genes are suggested flagella knockouts. Proteins in the *H. bluephagenesis* TD1.0 operon were determined first using the *E. coli* genome as reference, and next filling in open reading frames using genome annotation. All *E. coli* genes were discovered, although in a different order and in one large operon instead of 3 separate cassettes.

Genome mining for the sequenced genome was used to find all 50 structural and regulatory genes involved in flagella assembly reported in *E. coli* in the *H. bluephagenesis* TD1.0 genome (Figure 4). Orange genes in Figure 4 are suggested knockouts for *H. bluephagenesis* while green genes are other members of the flagella cassette. Notably, we found the FlhCD genes in *H. bluephagenesis* TD1.0, which code for a conserved class I transcriptional activator in other Gram-negative bacteria. This is predicted to transcribe gene class II operons FliA, FlhB, and FliL in *E. coli*.^30^ Genome curation revealed a single large operon consisting of 52 flagella and motility-related proteins as well as few smaller operons containing flagella-related genes (Figure 4). The open reading frames neighbouring these genes were annotated using BLASTp with default parameters to determine any relevant new genes absent in *E. coli* that are documented in other bacteria. For example, we identified the gene *flhF* in *Halomonas* that is reported to control flagella position in some organisms and be a flagella regulatory factor in others.^31^ The more completely annotated flagellar genes will be useful for future engineering efforts and knockout targets to reduce the energy waste related to flagellar assembly and rotation in an industrial setting.

## Discussion

Changes in environmental conditions can create shifts in metabolic flux to balance energy requirements for organisms under stress. For example, ^13^C tracing experiments show *C. salexigens*, a taxonomic neighbour to *H. bluephagenesis*, will produce excess metabolites such as acetate and pyruvate at low salt. This suggests its metabolism is not well-adapted to low osmolarity and will shift into overflow metabolism.^32^ In contrast, at high osmolarity, *C. salexigens* will have efficient energy usage and adapt its metabolism to support high metabolic flux to ectoine. Therefore, understanding the favoured resource allocation for a particular environment is important to further *H. bluephagenesis* TD1.0 industrial productivity.

### ABC transport pathway enrichment reveal salt tolerance strategy for H. bluephagenesis

The ABC transporters are among the top differentiated pathways between salt conditions, suggesting this pathway is involved in *H. bluephagenesis’* salt tolerance strategy. Different halophiles have their own preferred method to combat salt stress.^29^ Extreme halophiles such as archaea absorb KCl or other salts to balance osmotic pressure, while more moderate halophiles such as *H. bluephagenesis* are known to biosynthesize or import osmolytes.^33^ We found the ectoine synthesis pathway was upregulated as salt concentration increased, unsurprising as ectoine is a well-documented osmolyte biosynthesized *H. bluephagenesis*.^33^ However, other osmotic response mechanisms in *H. bluephagenesis* are less well-characterized. Our analysis indicates that glycine betaine/proline, glutamine, and trehalose/maltose ABC transporters are upregulated at high osmolarity, suggesting *H. bluephagenesis* TD1.0 might favour these osmoprotectants for import under osmotic stress. These less studied mechanisms for *H. bluephagenesis* TD1.0 to combat high salt are useful especially when targeting knock-down pathways to improve ectoine production.

Prior research reveals other *Halomonas* species adjust transport genes expression at different osmolarities. At high salt stress, *Halomonas alkaliphila* XH26 increased iron, phosphate, and glycine betaine ABC transporters along with ectoine synthesis,^34^ while *Halomonas* sp. AAD12 downregulated amino acid transport and increased proline concentration.^35^ Both trends are similar to our data and support our analytical strategy. *Chromohalobacter salexigens* transporters have highly acidic extracellular components compared with the non-halophilic bacterium *E. coli*,^36^ suggesting halophiles transporters themselves may function to combat salt stress due to their comparatively acidic extracellular components. Transporter enzyme engineering could be a strategy to maintain industrial titre at new salinities by fine-tuning the salt response, although further research into transporter acidity measurements is needed to confirm whether transporter regulation is tied to environmental acidity in *H. bluephagenesis*.

### Butanoate metabolism pathway enrichment indicates PHA is affected by salinity

Our findings suggest excess salt increases butanoate metabolism and therefore the production of PHA in *H. bluephagenesis*. The reason for butanoate metabolism downregulation at lower salt is unclear, as PHB in *H. bluephagenesis* is often considered to be a carbon source and storage and not involved in salt stress. In the archaeon *H. mediterranei*, a high salt concentration is suboptimal for growth but improves PHA production, while lower salt prevents PHA synthesis, suggesting other halophilic organisms use PHA production to combat salt stress.^37^ PHA itself may be an osmoprotectant for *H. bluephagenesis*, or a potential excess carbon storage molecule. These findings can be used to direct future engineering efforts for PHB, a highly valued industrial biodegradable plastics product.

### Pathway enrichment for RNA polymerase, ribosome, and quorum sensing

We found upregulation of transcription and translation in high/low salt was modulated by transcription factors and RNA-binding proteins, respectively. Previous research has theorized that at constant protein quantities, an increase in transcription will also increase the cost and precision of expression.^38^ It follows that genes both highly transcribed and translated are costly, imprecisely expressed, and likely very important. Stressed cells in a new environmental condition must search for an appropriate phenotype leading to much higher gene expression than in a stabilized environment.^39^ Under these suboptimal salt conditions, it is possible that the cell is poised to adapt to future environmental fluctuations and the high expression of ribosome and RNA polymerase is a stress response. Quorum sensing upregulation at high and low salt supports this idea, as quorum sensing allows bacteria to quickly communicate and adjust community-wide gene expression. Quorum sensing was linked to the osmotic stress response in the halophilic *Acinetobacter nosocomialis* bacteria,^40^ while the adaption of *Halomonas beimenensis* to salt was suggested to be dependent on genes relating to cell motility, quorum sensing, and transcription and translation.^41^ It is logical to conclude that in a higher stress environment, the combination of quorum sensing, RNA polymerase, and translation machinery would be upregulated to enable rapid adjustment to incoming stimuli. Therefore, we suggest RNA polymerase and ribosomal proteins are used as pre-emptive protection to anticipated environment changes and may not be specific to just the osmotic stress response.

### The flagellar assembly is a high-energy consumer in different salinities

We found that the flagella proteins were especially upregulated both at low- and high-salt conditions compared with the more growth advantageous LB60. Chemotaxis and motility are well-recognized responses in bacteria to hostile environments,^42^ with osmotic stress at low salt shown to induce flagellar biosynthesis and swimming in *Escherichia albertii*.^43^ Meanwhile, biofilming is a stress tool induced at high osmolarity in the halotolerant soil bacteria *Halomonas meridiana* and *Halomonas aquamarine*.^44^ Furthermore, flagella in multiple *Halomonas* species were shown to be energized by Na^+^ and H^+^ gradients, suggesting salt and pH may affect motility for certain species.^42^

The energy resource load required for flagella production and movement is considerable, as each flagellum is a complex machine consisting of more than 20 proteins and requiring more than 50 proteins for regulation and assembly (Figure 2). Moreover, the energy required to drive a flagellum motor is dependent on adenosine triphosphate (ATP) consumption. For example, flagella require ~2.3 × 10^5^ ATP molecules per beat, although the exact energy required varies by bacteria type.^45^ Whether flagellar assembly is a stress response or a consequence of overflow metabolism, in an industrial setting, the flagella are a waste of transcriptional, translational, and ATP expenditure. Should flagellar assembly be *H. bluephagenesis* TD1.0’s preferred use of energy resources in low-salt conditions, our results suggest that a genomic flagellum knockout has the potential to improve production at lower salinities.

### Flagellar assembly is complex with many possible targets for genomic engineering

The number and complexity of flagella-related genes is extensive, making a gene knockout strategy targeting flagella repression difficult without extensive engineering. Success in prior work for other organisms has been seen for knockout of the *FliA* operon, as the *FliA* sigma factor is predicted to bind to 22 gene promoters in *E. coli*.^46^ These class III genes include flagellar filaments, chemotaxis genes, and motor proteins needed to rotate the flagella.^47^ We suggest a *FliA* sigma factor knockout would lead to cells that still translate the flagellar hook and body proteins, but flagella will not fully assemble or rotate. Therefore, we propose FliA removal may decrease the expensive energy expenditure of the flagellar assembly while minimally tampering with the genome.

Alternative genetic engineering targets are the first genes synthesized in flagellar assembly and are required for flagellar swimming (FliC and FliF).^48^ Three *FliC* genes were identified in the *H. bluephagenesis* TD1.0 genome (Figure 4). Cells without FliC proteins partially form flagella but are limited in their ability to move and form biofilms.^49^ One *FliF* gene was annotated nearby genes *FliI, FliG*, and *FliH*, responsible for the ATPase that powers flagella rotation. Studies report that *FliF* deletion prevents *Salmonella* motility.^50^ We propose a *FliF* gene deletion would curtail synthesis of flagella proteins, while a *FliGHI* knockout could lower flagella energy expenditure. These minimal genome manipulations at a later stage would be good targets for removal to validate the omics study.

## Conclusions

Herein, we have presented a new web platform that facilitates analysis of the *H. bluephagenesis* genome for the determination of differentiated genes, proteins, and pathways between different growth conditions. Importantly, our interactive website allows scientists working in the field of synthetic biology to analyse and use omics data without requiring the knowledge of computational biology tools. Using this platform, flagellar assembly was discovered to be the third most differentiated area of metabolism after changes in ribosome and RNA polymerase synthesis, with a PCA loading of 0.2. Flagellar assembly was especially differentiating for *H. bluephagenesis* under different salinities. Microscopic studies showed 87% of cells swimming in low salt compared with around half of cells in higher salts, with low-salt cells travelling 1.8 times greater than the higher salt media. As we observed higher flagellar in a luminescent stain for low-salt conditions, it is reasonable that *H. bluephagenesis* synthesizes flagella to a greater extent in low salt. Therefore, targeting the elimination of functional flagella in *Halomonas* may be a useful way to improve growth and/or target secondary product formation under low-salt conditions.

Our new web platform is simple to update with new omics data from future experiments, which further improves its usefulness for a non-specialist user. We believe this tool will be useful to more rationally direct genomic engineering efforts and build understanding of this important industrially relevant organism.

## Supplemental Material

sj-docx-1-bbi-10.1177_11779322231171779 – Supplemental material for Online Omics Platform Expedites Industrial Application of Halomonas bluephagenesis TD1.0 Click here for additional data file. Supplemental material, sj-docx-1-bbi-10.1177_11779322231171779 for Online Omics Platform Expedites Industrial Application of Halomonas bluephagenesis TD1.0 by Helen Park, Matthew Faulkner, Helen S Toogood, Guo-Qiang Chen and Nigel Scrutton in Bioinformatics and Biology Insights
