# Supplementary material for: Online Omics Platform Expedites Industrial Application of Halomonas bluephagenesis TD1.0
Source: Bioinform Biol Insights. 2023 May 9;17:11779322231171779. doi: 10.1177/11779322231171779 (PMC10185862; doi:10.1177/11779322231171779)
Supplement: sj-docx-1-bbi-10.1177_11779322231171779 – Supplemental material for Online Omics Platform Expedites Industrial Application of Halomonas bluephagenesis TD1.0 [file sj-docx-1-bbi-10.1177_11779322231171779.docx]

# Supplementary Information

# On-line Omics Platform Expedites Industrial Application of *Halomonas bluephagenesis* TD1.0

**Helen Park^a,b^, Matthew Faulkner^a^, Helen S. Toogood^a^, Guo-Qiang Chen^b^ & Nigel Scrutton^a^**

^a^EPSRC/BBSRC Future Biomanufacturing Research Hub and BBSRC Synthetic Biology Research Centre SYNBIOCHEM, Manchester Institute of Biotechnology and Department of Chemistry, The University of Manchester, Manchester, UK M1 7DN

^b^Center for Synthetic and Systems Biology, School of Life Sciences, Tsinghua-Peking Center for Life Sciences, Tsinghua University, Beijing 100084, China

# Supplemental Figures

#
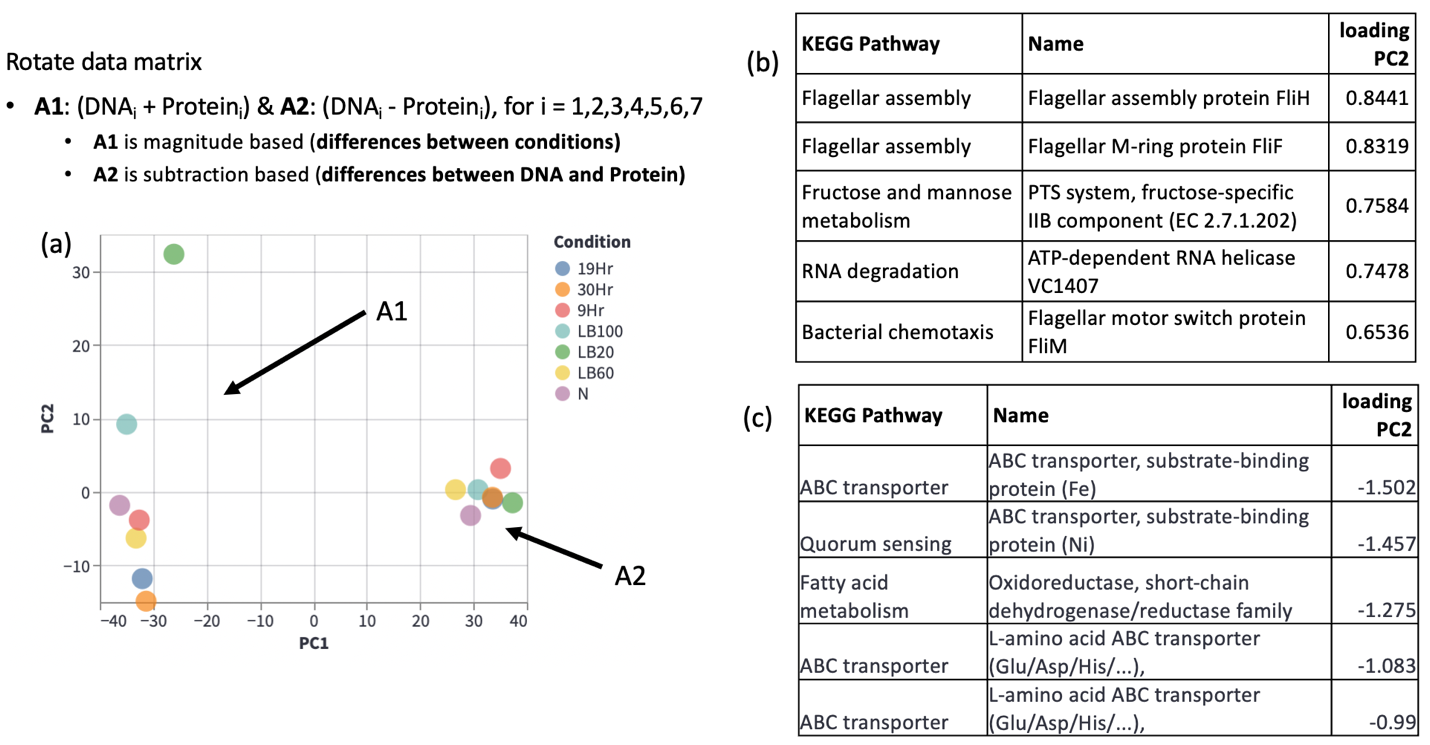


**Figure S1. Transformed PCA analysis, and most variable genes along PC2.** (a) Method used to rotate matrix and correct for any differences between proteomics and transcriptomics datasets. A1 are datasets with proteomics and transcriptomics added, enhancing any differences between conditions. A2 is subtraction, ameliorating differences between conditions. (b) Top 5 genes with high positive loadings in PC2. (c) Top 5 genes with high negative loading in PC2.

**
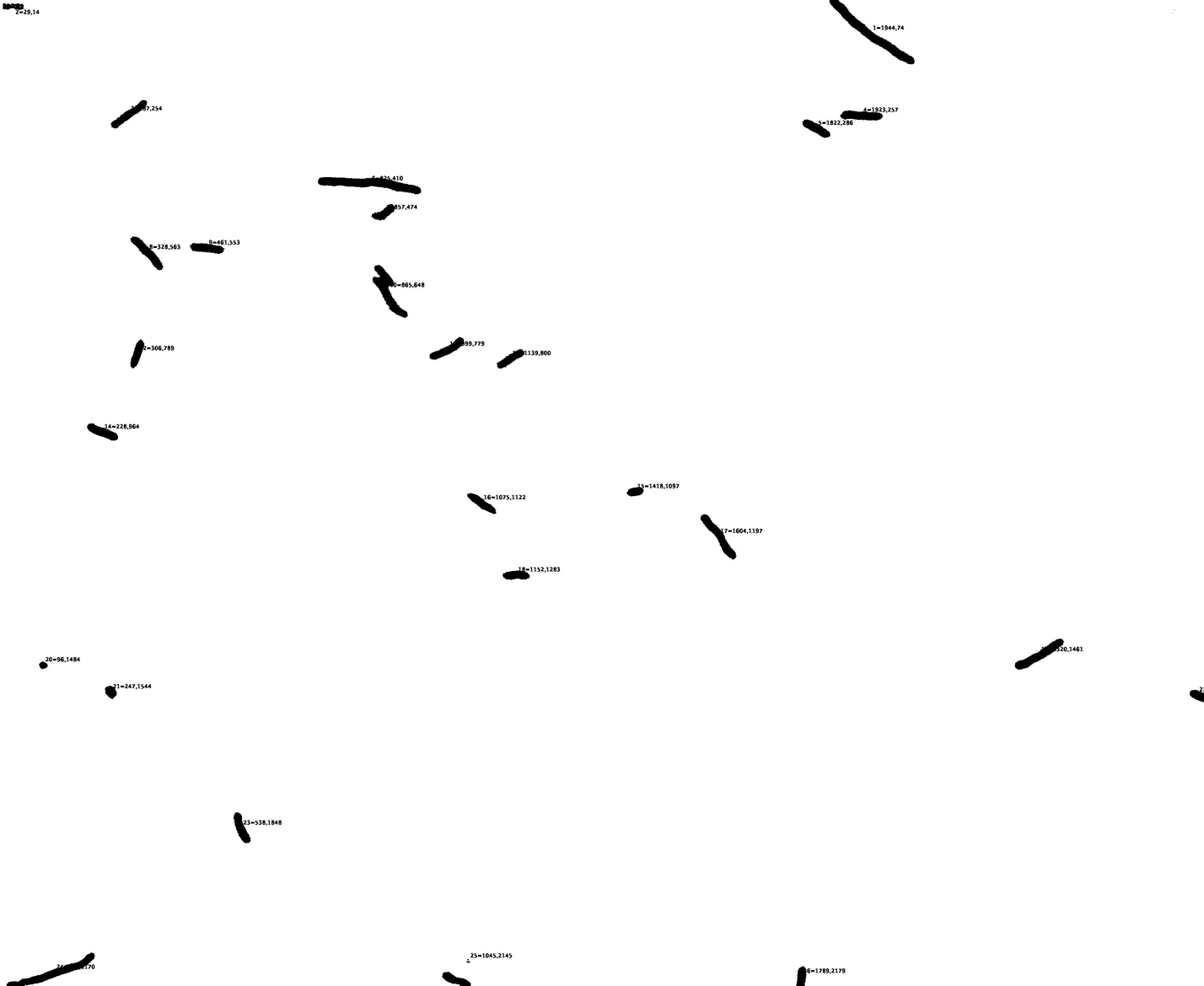
**

**Figure S2.** Cell tracking with Fiji, and morphology and biofilm formation differences under different salts. Example of final movie after Fiji processing, movie shows TD1.0 grown in LB20 at t=30. Black lines are cells, cells that are tracked have their position written in text, while cells not detected in Fiji have no text. **Double click the image to start the embedded movie.**


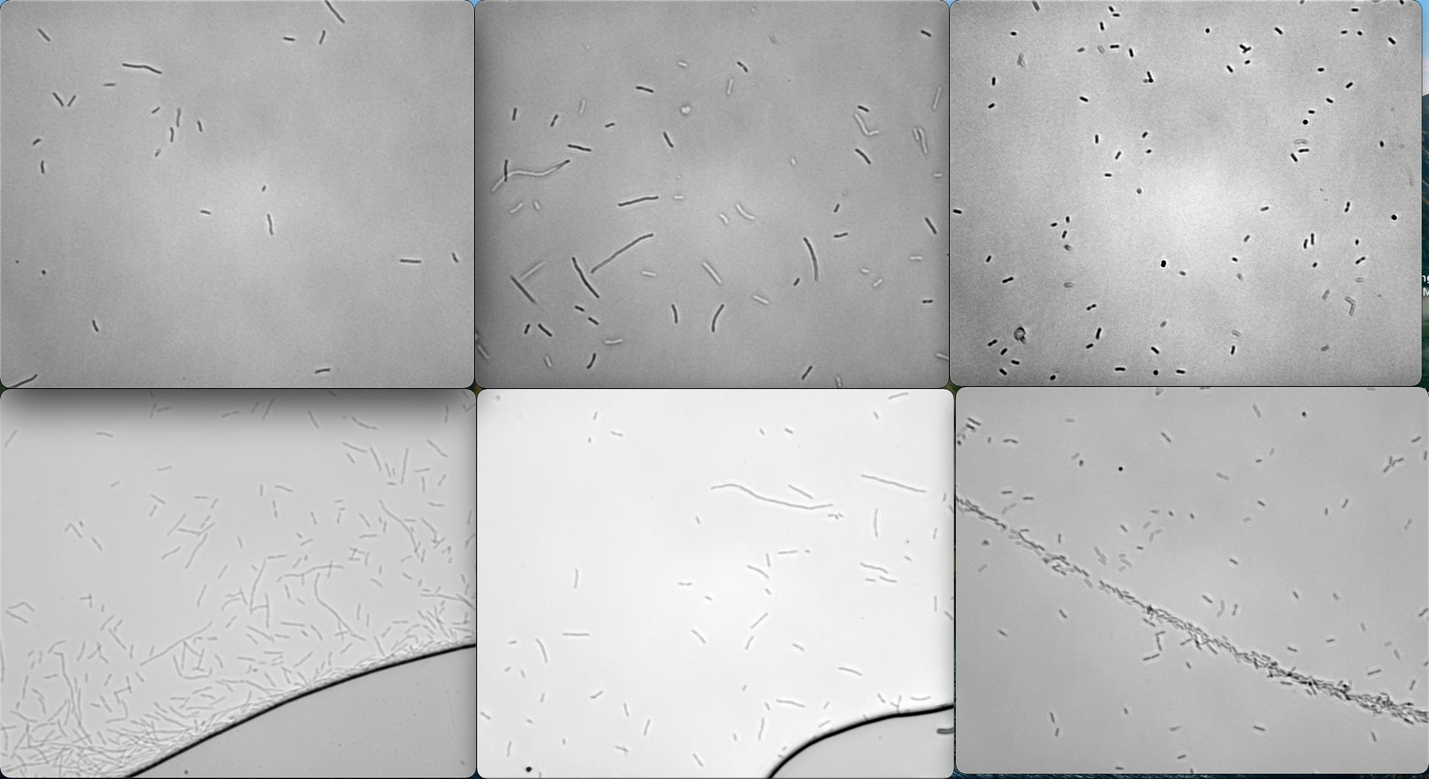


**Figure S3.** Cell tracking with Fiji, and morphology and biofilm formation differences under different salts. Example videos for TD1.0 cells tracked in each salt condition at t=30 (top row) and t=60 (bottom row.) Top row shows clear morphology differences between LB20, LB60 and LB100. Bottom row shows biofilm beginning to form for LB100 cells and LB20 cells migrating to the surface. At t=60 the coverslip is beginning to dry and therefore these time points are not used for quantification. **Double click the image to start the embedded movie.**
